# The effect of kidney transplantation on speckled tracking echocardiography findings in patients on hemodialysis

**DOI:** 10.15171/jcvtr.2018.14

**Published:** 2018-06-29

**Authors:** Sahand Hamidi, Javad Kojuri, Armin Attar, Jamshid Roozbeh, Alireza Moaref, Mohammad Hossein Nikoo

**Affiliations:** ^1^Cardiovascular Research Center, Department of Cardiovascular Medicine, Shiraz University of Medical Sciences, Shiraz, Iran; ^2^Student Research Committee, Shiraz University of Medical Sciences, Shiraz, Iran; ^3^Nephrology Division, Internal Medicine Department, Shiraz University of Medical Sciences, Shiraz, Iran; ^4^Shiraz Nephro-Urology Research Center, Shiraz University of Medical Sciences, Shiraz, Iran; ^5^Transplant Research Center, Shiraz University of Medical Sciences, Shiraz, Iran

**Keywords:** Speckle Tracking, Echocardiography, End-Stage Renal Disease, Renal Transplantation

## Abstract

***Introduction:*** Cardiac dysfunction is a major cause of morbidity and mortality in patients with end-stage renal disease (ESRD). Previous studies have shown that kidney transplantation can reverse some of the gross changes in the myocardial structure such as left ventricular ejection fraction (LVEF) and volumes. Whether kidney transplantation can reverse the subtle and early myocardial changes in ESRD patients who do not suffer from gross alternations in myocardial function is not yet studied. The aim of this study was to answer this question.

***Methods:*** We followed 25 patients with ESRD at baseline that all of them had a kidney transplant and were reassessed 1 month after the transplantation. Conventional and speckle tracking echocardiography (STE)was done at baseline and 1 month after kidney transplantation in patients.

***Results:*** LV hypertrophy was the most prevalent finding at baseline (58%), followed by diastolic dysfunction (53%). Kidney transplantation significantly improved the ejection fraction (EF) (treatment effect = 4.23 ± 2.06%; *P * = 0.046) and apical 4-chamber strain (treatment effect = -0.89 ± 0.37%; *P * = 0.021) in the patients. It also reduced the LV mass index (treatment effect = -73.82 ± 11.6; *P * < 0.001) and relative wall thickness (treatment effect = -0.056±0.023; *P * = 0.021). Other variables including global longitudinal strain and diastolic dysfunction were not improved significantly.

***Conclusion:*** STE may show early improvements in myocardial function 1 month after renal transplantation.

## Introduction

Cardiac disease is common in patients with end-stage renal disease (ESRD), and these patients have high morbidity and mortality often related to cardiac disease. In one report, myocardial infarction and cardiac arrest were reported in up to 39% of such patients.^1^ Gross alterations in the myocardial structure have been noticed in several studies. Left ventricular (LV) systolic and diastolic dysfunction had a prevalence of 38.4% in ESRD patients most of whom (82%) were asymptomatic.^2^ However, symptomatic heart failure in ESRD patients (ejection fraction [EF] less than 40%) is shown to be associated with lower survival.^3^ The most prevalent echocardiographic finding in ESRD patients is LV hypertrophy (56.9%) and most of these patients had a normal systolic function.^4,5^ Prevalence of valvular regurgitation like mitral regurgitation (MR) and aortic regurgitation (AR) was 2.7% and 1.3%, respectively.^4^ EF and LV dilation were the strongest predictors of survival in these patients.^3-5^

Echocardiography is a practical available tool for detection of these gross cardiac changes before and after renal transplantation.^4^ In addition, new echocardiographic techniques are now available that can detect subtle cardiac alteration, and may help implement preventive therapies to protect the heart from gross alterations. Tissue Doppler imaging is a method for accurate estimation of myocardial systolic function, diastolic velocities and deformation information which is independent on volume load and is a good tool for precise assessment of LV function before systolic and diastolic dysfunction by conventional echocardiography appears.^6,7^ There is evidence of tissue Doppler imaging changes after renal transplantations that could measure systolic and diastolic function at subclinical stages and showed improvement of LV function post-transplant.^8-11^

Speckle tracking echocardiography (STE) is a method for early LV function change assessment and could be used for longitudinal LV function impairment when circumferential LV shortening remains preserved in the early phase of cardiac damage by diseases.^12-15^ It is based on speckle (Speckles are spots that appear by the interaction of the ultrasound beam on the myocardial fiber) displacement analysis and is non-Doppler and non-angle dependent.^16^ STE application in the cardiac evaluation included hypertension-related concentric changes, subclinical LV function impairment in diabetes and coronary artery disease, cardiomyopathies (including uremic cardiomyopathy),^17^ valvular heart disease, heart failure evaluation, mechanical dyssynchrony and heart transplantation.^16^ In an animal study, Kramann and colleagues showed that STE parameters were the first parameters to decrease after a renal failure occurs and could predict cardiac mortality.^17^

The role of speckle tracking is not still evaluated in humans with ESRD. Therefore, we decided to conduct this study using conventional and STE indices before and 1 month after renal transplantation to show how and when LV function change occurs.

## Materials and Methods

### Patients

In this prospective study, 25 patients with ESRD who candidate for Kidney transplantation was included and was evaluated with echocardiography (conventional and STE) at baseline. Patients underwent kidney transplantation and a new echo 1 month after renal transplantation was performed on them to investigate the acute benefits of kidney transplantation on the heart. Each patient was aware of the target of echocardiography exam and aim of the study was explained to them. Inclusion criteria were being on hemodialysis, no previous coronary artery disease or valvular heart disease and not a previous transplant kidney recipient.

### Measurements

Blood Pressure (threshold of Hypertension considered ≥140/90 mm Hg), ESRD cause and duration of dialysis from the start and dialysis frequency extracted from patients’ records and echocardiography measurements based on ASE (American Society of Echocardiography) guideline on chamber quantification 2015^18^ and guideline on diastolic function assessment^19^ were evaluated on patients.

All echocardiographic evaluations were performed via General Electric’s (GE) E9 machine with a M5ScD probe with a bandwidth of 1.5-4.6 MHz 1 day after hemodialysis sessions to achieved relative dry weight while echo exams. Each patient by complete informed consent underwent two rounds of echocardiography by two expert operators who were unaware of treatment to reduce observer and interobserver variabilities. Reported data are a mean of these examinations.

Parameters in echocardiography examination included (1) LV morphology: Left ventricular end diastolic diameter (LVEDD), LV end systolic diameter (LVESD), LV end-diastolic volume (LVEDV), LV end systolic volume (LVESV), Posterior wall thickness (PWT), Inter-ventricular septal thickness (IVS), LV mass (LVM), LV mass index corrected by body surface area (LVMI), and Relative wall thickness (RWT). (2) LV systolic function: EF by Simpson method, Fractional shortening (FS). (3) Diastolic function: Early mitral inflow diastolic velocity (E), atrial contraction mitral inflow diastolic velocity (A), E/A ratio, tissue Doppler velocity at Interventricular septum (e¢), E/e¢ ratio. Cutoff values for abnormal diastolic function consider as e¢<7cm/s, E/e¢>15, E velocity >50 cm/s and E/A ≤0.8 or ≥2 according to ASE guideline 2016 for Diastolic function assessment.^19^ LV longitudinal speckle pattern also checked by STE Examination. Speckle tracking assessments were performed in apical long axis (ALAX), apical four chambers (A4C) and apical two-chamber (A2C) views by myocardial tracking and global longitudinal strain (GLS: mean of these measures) depicted as Bowl’s eye pattern. Strain rates were analyzed automatically by GE E9 Echocardiography machine software. The cutoff value for abnormal strain rates considered <-19% according to related articles.^20,21^ Fortunately, all of the patients were suitable for STE and good myocardial tracking was achieved.

### Statistical analysis

Data were entered into a statistical package for social sciences, version 17.0 (SPSS Inc., Chicago, IL, USA). Descriptive data were presented as mean, standard deviation, frequency, and percentage. Continuous variables were described as mean ± SD. *P* values less than 0.05 were considered statistically significant. For comparison of within-group analysis, paired t-test was used.

## Results

As shown in Table 1, the mean age of the patients was 44.64 ± 13.9 years. Most of the patients were male (84% of patients). Causes of ESRD were hypertensive nephropathy, diabetic nephropathy, autosomal dominant polycystic kidney disease (ADPKD), Alport syndrome, nephrotic syndrome, chronic glomerulonephritis and idiopathic or unknown causes were most prevalent in patients (40%) and hypertensive nephropathy (30%) and both diabetic nephropathy and unknown causes (25%) had the highest prevalence after that.

**Table 1:** Demographic data

**Variable**	**Percent, Mean ± SD**
Age (y)	44.64±13.91
Gender	84% Male, 16% Female
ESRD cause	40% idiopathic, 30% HTN
ESRD duration month	67.6±63
HD duration month	56.04±9.7
HD session/week	2.6±0.81

Abbreviations: HTN, hypertensive nephropathy, ESRD, end stage renal disease, HD, hemodialysis.

All the patients were on hemodialysis and those with peritoneal dialysis were not included in this study. Mean ESRD duration was 67.6 months. Mean hemodialysis session per week was about 2.6 sessions per week. Post-transplantation all of the patients were receiving cyclosporine and 88% were hypertensive.

In the baseline echocardiographic evaluations, LVH (LV mass index more than 116/m² in male and more than 96/m² in female) was the most prevalent echocardiographic finding present in 21 out of 25 patients (84%). In the LVH type, most of the patients were concentric hypertrophy (71.5%) with criteria of relative wall thickness (RWT) more than 0.42 and LVMI more than 116/m² in the males and more than 96/m² in the females. Eccentric hypertrophy (RWT less than 0.42 and LVMI more than 116/m² in males and more than 96/m² in female) was present in only 6 patients. Diastolic dysfunction (tissue Doppler e¢ velocity less than 0.07 m/s measured at interventricular septum) was the second with 48% prevalence. Diastolic dysfunction persisted 1 month after renal transplantation in 44% of the patients. Prevalence of Systolic Dysfunction with criteria of EF by Simpson method less than 40% were 8%. Table 2 displays the measurements at baseline.

**Table 2:** Comparison of the changes in echocardiographic measures after one month of transplantations in patients

**Variable**	**Baseline measures**	**1m Later measures**	**Difference**	***P*** ** value**
RWT	0.43±0.11	0.35±0.08	-0.08±0.09	0.000
LVMI (g/m^2^)	148.5±50.2	92±32.5	-56.5±49	0.000
EF (%)	61.77±13.7	64.8±8.6	3.03±6.7	0.033
FS (%)	32.3±9.4	36.8±7.7	4.5±8.3	0.012
e′ (m/s)	0.08±.02	0.08±0.02	0.003±0.01	0.345
E/e′	10.8±3.7	9.9±2.37	-0.91±2.5	0.083
PLAX strain (%)	-18.2±3.9	-18.6±3.5	-0.4±1.9	0.304
A4C strain (%)	-18.2±2.9	-18.9±3.4	-0.75±1.6	0.029
A2C strain (%)	-18.8±3.4	-18.5±3.1	0.22±2.35	0.639
GLS (%)	-18.4±3.11	-18.7±3.2	-0.32±1.48	0.277

Abbreviations: RWT, relative wall thickness; LVMI, left ventricular mass index; EF, ejection fraction; FS, fractional shortening; PLAX, parasternal long axis; A4C, apical 4-chamber; A2C, apical 2-chamber; GLS, global longitudinal strain.

The difference between baseline and 1-month later measurements of echocardiographic variables was calculated in patients and compared, as shown in Table 2. RWT and apical four-chamber strain were decreased significantly in 1 month after kidney transplantation. Transplantation also led to an improvement in EF and a reduction in LVMI. Other variables showed no significant difference.

## Discussion

Here, we have shown that subtle and gross changes in myocardial function assessed by conventional and STE are improving with kidney transplantation in patients with ESRD.

Pakfetrat et al showed in a previous study^6^ evaluating 1354 ESRD patients from 2000 to 2010; LVH was the most prevalent echocardiography finding (47.5%) and atrial dilation, diastolic dysfunction, dilated end diastolic dimension were followed next, and systolic dysfunction was the least prevalent (18.5%). Older age, male gender, longer duration of hemodialysis and anemia correlated with echocardiographic abnormalities and hypocalcemia was associated with atrial and LV End diastolic dilation. Each gram drop of Hemoglobin in ESRD patients showed to be correlated with the rise of LV mass about 10 g/m.^4^ In our study, LVH was the most prevalent echocardiographic measure (58%), a finding which is inconsistent with previous findings. Post Renal-transplantation LV mass may decrease significantly during 3 months, and after 12 months LV mass became normal in 23% of cases as shown in one study.^22^ Our study results showed that LV mass regression can start earlier than 3 months after renal transplantation (1 month).

In the study of Dȩbska-Ślizień et al,^23^ improvement of EF and LV systolic indices was shown after one year of transplantation. In the study of Dzemidzic et al,^22^ 1 year after renal transplantation 63% of transplanted patients showed normal EF. Our results also showed that EF would improve with transplantation even within 1 month of transplantation. The effect that may partially attribute to decrease volume overload in patients after transplant.

In the study of Dȩbska-Ślizień et al,^23^ diastolic dysfunction persisted after kidney transplantation. The authors attributed this finding to cyclosporine usage, hypertension, and myocardial fibrosis. Other studies^24^ have shown that renal transplantation can improve the LV function and LVH by removal of negative chronotropic and inotropic molecules existed in the serum of ESRD patients and longer duration of hemodialysis pre-transplant cause lesser improvement of LV function post-transplant. In agreement with previous studies, we also noticed that diastolic dysfunction which was present in 48% of patients’ pre-transplant, persisted in post-transplant for a 1-month period in 44% of patients although LVMI had improved with a mean 55 g/m² decrease. Therefore, the persistence of diastolic dysfunction could not be simply explained by the persistence of LVH but may be related to other factors such as cyclosporine usage and persistent Hypertension. The study of Rakhit et al demonstrated that e¢ velocity in tissue Doppler imaging can predict mortality and major cardiovascular event independently, and improvement of e¢ velocity noted during 4 years after renal transplantations from 5.6 to 6.5 cm/s.^9^ Our result revealed no significant rise in e¢ Doppler velocity 1 month after renal transplantation. One possible explanation for this failure to rise may be the shorter duration of the post-transplant evaluation period in our study. Various studies evaluating diastolic LV function changes post-renal transplantations showed variables results that may be due to angiotensin-converting enzyme (ACE) gene polymorphism, AV fistula patency, immunosuppressant drugs, control of Hypertension, graft function, normal hemoglobin level and infection and rejection status. The lower level of immunosuppressant drug and sirolimus usage is associated with lower prevalence of diastolic dysfunction and higher LVH regression.^8,25-28^

To the best of our knowledge, few previous human studies evaluated the effect of kidney transplantation on the speckle tracking parameters. In an animal study, Kramann and colleagues showed that STE was the first parameter which decreased after the renal failure occurred and could predict cardiac mortality.^17^ For this reason, we evaluated STE parameters at baseline and after 1 month due to the capability of earlier improvement and deterioration demonstration. In other cardiac situation such as ischemic heart disease, it was shown that speckle tracking could show alteration in LV function months before conventional echocardiography measure changes developed. Our Study results showed that apical 4-chamber strain is the only STE parameter that changed significantly after transplantation.

Our study had some limitations. Lack of a significant difference in some variables such as global longitudinal strain may be related to our sample size and higher sample sizes may reveal significant differences. A short time after transplantation may also affect the other variables to change because other researchers showed the improvements months and years after transplantation. Lack of drug usage associations with echocardiography measures was another limitation of our study, because drug used by patients may influence echocardiography parameters.

It can be concluded that cardiac dysfunction caused by ESRD and uremic cardiomyopathy may be improved by renal transplantation even as early as 1-month post-transplantation. LVMI, apical 4-chamber strains are two useful parameters to follow gross and subtle improvements in the myocardial function and structure after transplantation. Further studies are needed to compare echocardiographic changes between hemodialysis and peritoneal dialysis patients. Studies for determination of molecules responsible for cardiac depression in hemodialysis patients which has been removed by transplant are also needed to be conducted for implementation of better preventive strategies.

## Ethical approval

This study was approved by the ethics committee, Shiraz University of Medical Sciences, Shiraz, Iran (IR.SUMS.med.REC.1394.S15). Informed consent forms were signed by patients.

## Competing interests

None.

## Acknowledgements

The present article was extracted from the thesis written by Sahand Hamidi M.D and financially supported by Shiraz University of Medical Sciences grants No. 6499.
